# Outcomes and Complications of Posterior Fossa Surgery in Sitting Versus Park-Bench Positions

**DOI:** 10.3390/medicina60111855

**Published:** 2024-11-12

**Authors:** Oana Maria Radu, Georgeta Magdalena Balaci, Daniel Corneliu Leucuța, Vlad Ioan Moisescu, Cristina Munteanu, Ioan Ștefan Florian

**Affiliations:** 1 Department of Neurosurgery, “Iuliu Hațieganu” University of Medicine and Pharmacy, Victor Babeș Street, No. 8, 400347 Cluj-Napoca, Romania; raduoanam@yahoo.com (O.M.R.); stefanfloriannch@gmail.com (I.Ș.F.); 2 Clinic of Neurosurgery, Cluj County Emergency Clinical Hospital, Victor Babeș Street, No 43, 400012 Cluj-Napoca, Romania; 3 Department of Medical Informatics and Biostatistics, “Iuliu Hațieganu” University of Medicine and Pharmacy, Pasteur Street, No 6, 400349 Cluj-Napoca, Romania; 4 The Regional Institute of Gastroenterology and Hepatology Octavian Fodor, Croitorilor Street, No. 19, 400394 Cluj-Napoca, Romania; vlad.moisescu94@yahoo.com (V.I.M.);

**Keywords:** hemodynamic implications, venous air embolism, sitting position, park-bench position, EtCO_2_, pneumocephalus, postoperative hematoma

## Abstract

*Background/Objectives*: Patient positioning during surgery can influence intra- and postoperative complications. Therefore, we assessed the impact of the sitting and park-bench positions on anesthetic parameters and complications in neurosurgical patients. *Methods and Patients*: For this retrospective study, 314 adults who underwent neurosurgical procedures for posterior fossa pathologies were divided into two groups: sitting (*n* = 231) and park-bench (*n* = 83). The following data were collected, monitored, recorded, and compared: age, sex, tumor type, surgical approach, cardiovascular and respiratory complications, and postoperative surgical complications. The association of hypotension with the position was further investigated through multivariate logistic regression models by adjusting for CO_2_ decrease, desaturation, and documented gas embolism. *Results*: The average age was significantly lower in the sitting group (55 years, interquartile range (IQR) = 43–63; female proportion = 59.74%) than in the park-bench group (62 years, IQR = 45–74; female proportion = 57.83%) (*p* < 0.001). Cerebellopontine angle tumors were detected in 37.23% of the patients who underwent an operation in the sitting position and in 7.26% who underwent an operation in the park-bench position (*p* < 0.001). Patients in the sitting position had significantly greater anesthetic complication (91.77% vs. 71.08%, *p* < 0.001), hypotension (61.9% vs. 16.87%), and >2 mmHg CO_2_ decrease (35.06% vs. 15.66%, *p* < 0.001) incidences. Hypoxemia and death occurred more frequently in the park-bench group (8.43% vs. 1.73% and 6.03% vs. 1.3%, respectively). *Conclusions*: Compared with the park-bench position, the sitting position was associated with a greater specific anesthetic complication incidence and lower postoperative mortality rate, indicating a need for careful risk-benefit assessment when selecting each individual patient’s surgical position.

## 1. Introduction

Neuroanesthesia involves the clinical management of patients who are undergoing surgical procedures that require complex positioning. While there are several positions available for posterior fossa surgery, including prone and supine with head rotation, the focus of this study was limited to the sitting and park-bench positions due to their specific relevance and prevalence in clinical practice. These two positions are commonly associated with distinct anesthetic and surgical challenges, which warranted a detailed comparison. The choice of position varies widely from neurosurgeon to neurosurgeon on the basis of experience and skill, patient posterior fossa pathology, associated comorbidities and, not the last resort, the tradition of the neurosurgical department. Our center is the only one in Romania that uses the sitting position on a regular basis, allowing favorable circumstances for comparing different patient positions during posterior fossa surgery and indicating one of the novel aspects of the present study. The aim of intraoperative anesthesia monitoring is to monitor vital functions in real time, allowing for rapid and precise adjustments of anesthetic management, thus helping ensure safe invasive medical intervention for the patient.

The benefit of the sitting position for both neurosurgery and neuroanesthesia is still being discussed. The sitting position provides optimal exposure for surgery, decreases intracranial pressure, improves cerebral venous drainage, reduces the risk of bleeding, provides good access to the patient’s thorax and airway, allows better intraoperative neurophysiological monitoring of cranial nerves (allowing better intraoperative preservation of nerve function than other positions), and improves pulmonary ventilation because of lower airway pressure. However, the sitting position is associated with certain complications, such as hemodynamic instability, peripheral nerve injury, venous air embolism (VAE) and its secondary complications, intracranial hypotension, pneumocephalus, tetraplegia, and tongue and laryngeal edema [1].

In neurosurgery, the park-bench position is a commonly used position in posterior fossa tumor surgery and involves lateral stabilization of the patient. Although it may offer certain benefits in terms of surgical access, this position is generally preferred because of the low risk of developing VAE. However, this position is associated with numerous hemodynamic effects; for example, blood may accumulate in the dependent lower extremities, causing a reduction in venous return and the occurrence of arterial hypotension. Additionally, the inferior vena cava may be partially or completely obstructed by hip flexion, which may lead to venous stasis of the lower extremities. This position may also result in an altered ventilation/perfusion ratio and decreased pulmonary compliance and functional residual capacity in anesthetized patients [2]. Airway visualization and access are difficult, and the risk of atelectasis and pneumonia increases owing to the prolonged time spent under anesthesia in this position. Another undesirable effect of this position is vision loss and brachial plexopathy. These complications can significantly influence neurosurgical outcomes, with a major impact on recovery and the general condition of neurosurgical patients.

Prior to choosing the position for the neurosurgical procedure, the following factors should be considered: absence of absolute contraindications, risks vs. benefits, and comfort level regarding the procedure, both for the surgeon and the anesthesiologist, so that communication is constant and immediate during surgery [3].

The physiological consequences of the positioning choice involve cerebral hemodynamics and intracranial pressure, which are closely related to the cardiorespiratory implications of patient positioning [4]. Both the sitting and the park-bench positions may lead to cardiovascular and pulmonary changes.

Although numerous studies have investigated the effects of the sitting positions during posterior fossa surgeries [5,6,7], there is a notable lack of comprehensive analyses comparing this position with the park-bench position. Given the complexity of posterior fossa pathologies and the potential for various intra-anesthetic complications during surgeries, it is crucial to evaluate both positions systematically. A comparative study is necessary to provide evidence-based recommendations for surgeons and anesthesiologists to optimize patient safety and outcomes.

## 2. Methods and Patients

### 2.1. Ethical Considerations

The present study was carried out in accordance with the regulations of the 1964 Declaration of Helsinki. Since this was a retrospective study, the consent of the Ethics Committee was not necessary, but patients or their legal representatives provided written consent for anesthesia and neurosurgical procedures.

This section was drafted with the support of a language model, ChatGPT, used to generate the initial draft. Subsequently, the authors verified, edited, and reviewed the entire content to ensure the accuracy of the section, in accordance with academic and professional standards.

### 2.2. Study Design and Setting

This retrospective cohort study included 314 adult patients who were hospitalized in the Neurosurgical Department of Emergency Clinical Cluj County Hospital between January 2015 and December 2021 and who were diagnosed and underwent an operation for posterior fossa pathologies.

### 2.3. Participants

For the purpose of our study, the patients were divided into two groups: patients who underwent an operation in the sitting position (*n* = 231) and patients who underwent an operation in the park-bench position (*n* = 83).

### 2.4. Inclusion Criteria

Patients aged 19 to 89 years and classified as having an American Society of Anesthesiologists (ASA) score of I, II, or III were enrolled for posterior fossa surgery performed in either the sitting or park-bench position under general anesthesia.

The exclusion criteria were as follows: (1) patients classified as ASA score IV or those with systemic diseases unrelated to the central nervous system (CNS), such as cardiac, renal, or respiratory conditions; (2) patients who underwent surgeries conducted in positions other than sitting or park-bench; and (3) patients with incomplete medical records.

## 3. Variables

The data collected from medical records included patient characteristics; tumor type; anesthesia technique; monitoring methods used; documentation of intraoperative VAE; hemodynamic, cardiovascular, and pulmonary changes occurring during neurosurgical intervention; events occurring immediately after surgery; total time (minutes) of surgery and anesthesia; and the total number of days spent in the intensive care unit or neurosurgery unit.

### 3.1. Patient Positioning

In the sitting position, the head was fixed in a Mayfield head clamp and slightly flexed, the torso was supported and slightly inclined to avoid hypotension, the lower limbs were slightly flexed at the knee and rested on a sponge support to prevent elongation of the sciatic nerve, and the upper limbs were positioned on a support at the elbow level.

For the park-bench position, the patient was positioned in the lateral decubitus position. Similar to the sitting position, the head was fixed in the metal frame, maintaining a neutral alignment between the shoulders and the neck to prevent compression of the vertebral arteries or jugular veins. The torso was supported by cushions or special support to prevent slipping, and the lower limbs were positioned to avoid peripheral nerve compression; the knee of the upper leg was slightly flexed and rested on a sponge support to prevent excessive strain on the sciatic nerve. The upper arm rested on an arm support at the trunk level, whereas the lower arm was supported on a sponge support with an axillary roll to avoid brachial plexus paresis.

In addition, because of the risk of hypothermia, active warming measures were taken (warmed blanket, warmed fluids). Deep vein thrombosis prophylaxis was performed with compression stockings or pneumatic compression devices. Prevention of pressure ulcers was performed by using gel cushions and frequently adjusting the position when necessary.

### 3.2. Anesthesia Technique

Midazolam was used as premedication when anxiety was present, and Xyline 1% was administered to decrease reflexes during intubation, depending on the anesthesiologist’s preference. Fentanyl or sufentanil was used to reduce the response to intubation, and propofol was used as a hypnotic agent. Atracurium or Esmeron was administered depending on comorbidities. All patients were intubated with a flexometallic tube. Anesthesia was maintained with inhaled sevoflurane, and in patients with intracranial hypertension, total intravenous anesthesia with BIS and TOF monitoring was carried out according to neurophysiological monitoring.

### 3.3. Monitoring of Intraoperative Anesthesia

(a) Hemodynamic monitoring included heart rate monitoring, 5-lead Electrocardiogram (ECG) for early detection of arrhythmia, and continuous noninvasive (blood pressure cuff) or invasive (radial artery cannulation on the arm accessible to the anesthesia team) blood pressure monitoring.
(b) Respiratory monitoring included capnography to monitor ventilation and detect early changes in CO_2_ levels, including early signs of VAE; oxygen saturation (SpO_2_) and arterial blood gases (occasionally) were also monitored to assess ventilatory and acid-base status.
(c) Central venous pressure monitoring was performed via a central venous catheter inserted in the right subclavian vein or internal jugular vein, which is essential for volemic resuscitation, vasopressor treatment, and, owing to the risk of developing VAE, air aspiration from the right atrium in the case of VAE occurrence.
(d) Neurological monitoring included somatosensory and motor-evoked potentials (SSEPs, MEPs) in patients with cerebellopontine angle tumor formations and brainstem or craniocervical junction tumors.
(e) Body temperature was monitored.
(f) Diuresis monitoring included bladder catheterization.

After surgery, patients were monitored in postoperative intensive care.

### 3.4. Statistical Analyses

Qualitative data are presented as counts and percentages. Normally distributed quantitative data are presented as the means and standard deviations (SDs). Nonnormally distributed quantitative data are presented as medians and quartiles 1 and 3. Comparisons between two independent groups were performed via Fisher’s exact test for qualitative data and via independent-sample *t* tests (normally distributed data) or Wilcoxon rank-sum test (nonnormally distributed data) for quantitative data. For all the statistical tests, a 0.05 significance threshold was used. All the statistical tests were two-sided. A multivariate logistic regression model was constructed to evaluate the association between hypotension and surgical position while adjusting for the potential confounding effects of decreased CO₂ levels, desaturation, and documented VAE and estimate the odds ratios (ORs) and corresponding 95% confidence intervals (CIs) for each predictor variable. Prior to model fitting, the assumptions of logistic regression were assessed, which included checking for multicollinearity among the predictor variables via variance inflation factors (VIFs), ensuring linearity in the logit for continuous predictors. The goodness-of-fit of the final model was assessed via the Hosmer–Lemeshow test.

All analyses were performed in the R environment for statistical computing and graphics (R Foundation for Statistical Computing, Vienna, Austria), version 4.3.2 (R Core Team. R: A Language and Environment for Statistical Computing. R Foundation for Statistical Computing: Vienna, Austria; 2024).

## 4. Results

The present study included 314 patients divided into two groups—sitting and park-bench position (Table 1): 231 patients were in the sitting group, and 83 patients were in the park-bench group. All patients who met the eligibility criteria were included in the study, and no patients were lost to follow-up. The participants’ mean age was 55 years (IQR: 43–63) in the sitting group and 62 years (IQR: 45–74) in the park-bench group. Most participants in both groups were female (59.74% in the sitting group and 57.83% in the park-bench group).

In terms of the clinical variables, the surgical approach used varied significantly between the two groups. The retrosigmoid approach (65.8%) was the most common approach in the sitting group, whereas in the park-bench group, the most common approach was the unilateral suboccipital approach (69.8%). A total of 91.77% of the patients in the sitting group experienced anesthesia complications, whereas 71.08% of those in the park-bench group experienced complications (*p* < 0.001). Hypertension was present in 51.95% of the patients in the sitting group and in 39.76% of the patients in the park-bench group, with a statistically significant difference (*p* = 0.002). Hypotension was more common in the sitting group (61.9%) than in the park-bench group (16.87%). Heart rate oscillations were present in both groups but in different manners: bradycardia was predominant in the sitting group (38.1%), whereas tachycardia was frequently present in the park-bench group (21.69%). The association of hypotension with position was further investigated through multivariate logistic regression models by adjusting for CO_2_ decrease, desaturation, and documented gas embolism (Table 2). After adjustment for the other variables, the risk of developing hypotension remained significantly associated with the position; the odds of developing hypotension were 7.25 higher for the sitting group than for the park-bench group (*p* < 0.001). Moreover, the odds of hypotension were 3.37 higher for those who had CO_2_ decreases of >2 mmHg (*p* < 0.001).

Vasoactive treatment was administered to a greater percentage of the patients who underwent an operation in the sitting position (36.8%), whereas 42.17% of patients in the park-bench group required volemic therapy. Several intraoperative variables significantly differed between the two groups: CO_2_ decrease (35.06%) and VAE (10.39%) occurred in the sitting group, and hypoxia (8.43%) occurred in the park-bench group. A total of 8.43% of patients who underwent an operation in the park-bench position required blood transfusions, whereas 2.6% of those who underwent an operation in the sitting position did. In terms of immediate postoperative extubation, anesthesia duration, and neurosurgery duration, the differences between the two groups were not statistically significant. However, the postoperative ventilation time was longer in the park-bench group by up to 5760 ± 719.69 min.

Postoperative complications included postoperative hematoma (5.63% vs. 7.23%), pneumocephalus (5.63% vs. 3.61%), hydrocephalus (4.33% vs. 4.82%), total facial paralysis (3.03%, found only in the sitting group, in cases of cerebellopontine angle tumors), and ischemia (0.87% vs. 1.2%), with no statistically significant difference between the two groups. The number of recorded deaths was slightly greater in the park-bench group (6.03%) than in the sitting group (1.3%).

## 5. Discussion

The present study included 314 patients with posterior fossa tumors, of whom 231 underwent surgery in the sitting position and 83 in the park-bench position. The patients were observed over 6 years, offering the opportunity to highlight the cardiovascular and respiratory implications in relation to intraoperative positioning. Both the sitting and park-bench positions show cardiorespiratory changes dependent on VAE, as well as changes independent of its occurrence. For both positions in question, we analyzed the major monitoring parameters, including both hemodynamic and respiratory variables. Patients in the sitting position had a significantly greater incidence of anesthetic complications and hypotension. Hypoxemia and death occurred more frequently in the park-bench group.

The most common causes of hemodynamic and respiratory instability during posterior fossa surgery are anesthesia induction, patient positioning, brainstem and cranial nerve manipulation, VAE, and rapid and severe bleeding. The response to these changes translates into hypertension, hypotension, tachycardia, bradycardia [7], hypercapnia, hypocapnia, and hypoxia.

In both the sitting position and the park-bench position, blood pressure may increase for various reasons related to anesthesia and positioning, as well as for each individual, depending on associated comorbidities. Various studies have shown that changing a patient’s position is associated with numerous cardiovascular changes [8].

Hypertension occurrence is one of the variables analyzed in the present study. The percentages varied from one position to another: in the sitting position, 51.95% of patients had hypertension, whereas in the park-bench position, 39.76% had hypertension. The variability of above-normal blood pressure values was observed, particularly during positioning as well as after positioning. In both positions, hypertension episodes may lead to worsening of cerebral edema and increase the risk of intraoperative bleeding. In our study, pain and stress were associated with increased blood pressure in both the sitting and park-bench positions. Pain occurs following cranial clamping, incisions, and dura mater opening and has stimulatory effects that may lead to hypertension and tachycardia. Anesthetic management is used to suppress the pain response both by injecting local anesthetic at the insertion site of the fixation device and by supplementing intravenous analgesia. Other causes leading to increased blood pressure were associated with each patient’s individual pathology, such as chronic arterial hypertension, certain drugs used in neurosurgery (corticosteroids), and secondary complications such as infections or cerebrospinal fluid (CSF) disturbances that may trigger a systemic response and lead to intraoperative hypertension.

In our study, arterial hypotension was considered if there was a decrease of more than 20% in the systolic blood pressure of the patient. In the sitting position, more than half of the patients had arterial hypotension (61.9%), while the percentage was significantly lower (16.87%) for the park-bench position. Hemodynamic instability does not always occur with the use of the sitting position. It has been demonstrated similar hemodynamic instability between patients in the prone and sitting positions [9].

Compared with another study published by Sonari Kore et al., in 2016 [8], where the occurrence of arterial hypotension was also observed during positioning, in our study, in both the sitting and park-bench positions, arterial hypotension was observed after anesthetic induction and until approximately 60 min after patient positioning. Blood pressure returned to normal afterward. An important aspect to consider and one frequently encountered in our practice is transient arterial hypotension, which is encountered later during neurosurgical intervention without a specific clinical cause; these findings were reported by the Helsinki team [10] and by Dallier, F. and Di Roio, C. [11]. Other predictive factors for the occurrence of orthostatic hypotension are as follows: antihypertensive medication and beta-blocker treatment, Parkinson’s disease status, diabetes mellitus status [12,13,14,15,16], and degree of dehydration.

In addition to the fluids administered for maintenance and observed losses, the patients in whom arterial hypotension was not transient required a combination of colloid and vasopressor treatment. In the sitting position, colloids were necessary for 25.54% of patients, and vasopressor support was necessary for 36.8% of patients, whereas in the park-bench position, the need for colloids occurred in 42.17% of patients, and the need for vasopressor treatment was much lower—18.07%. The difference in percentages between the two positions and the decision to administer colloid vs. vasopressor as first-line treatment for hypotension was determined by the anesthesiologist and varied depending on the patient’s response.

Heart rate variability depends on humoral mechanisms, cranial nerve manipulation, and the postural stress response [4].

In our study, tachycardia was present in 13.85% of the sitting position cases and 21.69% of the park-bench position cases. The presence of tachycardia was not only associated with pain-induced hypertension and VAE incidence but also with cranial nerve manipulation.

Bradycardia occurred in 38.1% of the cases in the sitting position and in 15.66% of the cases in the park-bench position. Bradycardia was associated with cranial nerve manipulation and was resolved in many cases after the surgeon was alerted and stopped the maneuver. Some of the patients also required atropine: 25.11% of those in the sitting group and 8.43% in the park-bench group.

In the case of the sitting position results, both tachycardia and bradycardia following tumor and cranial nerve manipulation were comparable with the results of a study published in 1976 by Albin, M.S. et al., in which tachycardia occurred in 23% of patients (13.85% in the present study), and bradycardia occurred in 25% of patients (38.1% in the present study) [7].

A frequently encountered complication in the sitting position is VAE. In our study, documented VAE occurred in 10.39% of the patients. VAE occurrence is frequently encountered when a negative gradient is created between atmospheric pressure and venous blood or when the cephalic extremity is located more than 20° above the heart [17]. Subatmospheric pressure in noncollapsing cerebral veins facilitates the entry of air; thus, VAE can induce right ventricular failure, pulmonary edema, and acute respiratory distress syndrome [18].

A wide range of VAE occurrence incidents (1.6% to 76%) are reported in the literature, and the majority of reported VAEs appear to be clinically irrelevant [19].

Because of the risk of VAE occurrence, it is vital to exclude patent foramen ovale in patients with an additional risk of paradoxical gas embolism due to a right-to-left shunt in the systemic circulation [19,20].

In this study, we used capnography to detect VAE by continuous monitoring of EtCO_2_ in parallel with hemodynamic parameters. Compared with a study published in 2011 by Ozlem Korkmaz Dilmen et al. [21], VAE diagnosed via EtCO_2_ had a 20.4% incidence in the adult population, a higher percentage than that in our study, where VAE was present in only 10.39% of cases. The results where VAE is detected through EtCO_2_ also appear in a study published by Konard et al. [22]. Although capnography is a less sensitive method for diagnosing VAE than transesophageal echocardiography and precordial Doppler ultrasound are, the use of capnography combined with the experience of the anesthesiologists and neurosurgeons was demonstrated to be useful for detecting VAE, making it an effective diagnostic tool with a low complication rate.

VAE without prompt initiation of treatment could be fatal for patients. Prompt treatment of VAE requires good communication between the anesthesiologist and the neurosurgeon. In our practice, when a drop of more than 2 mmHg of EtCO_2_ is observed on the monitor, in parallel with a drop in blood pressure and tachycardia onset, the surgeon is alerted and takes action by covering the operating field with saline-soaked compresses. Simultaneously, 100% oxygen is immediately administered, jugular vein compression is performed, air is suctioned through the central venous catheter, arrhythmias are treated, volemic resuscitation is performed, and vasopressor support is offered.

In the sitting position, three deaths occurred (1.3%), whereas in the park-bench position, five deaths occurred (6.02%). In the sitting position, the deaths were not VAE-related. A previous study reported a 1% mortality rate due to VAE [23].

EtCO_2_ values and capnography are useful tools in VAE diagnosis, as they are convenient and practical methods, albeit relatively insensitive compared with other monitoring techniques. Although these markers aid in VAE diagnosis, CO_2_ analysis from a single breath provides real-time information on CO_2_ production and elimination, metabolism, circulation, and ventilation [24]. Manipulating CO_2_ levels can help reduce cerebral blood flow and intracranial pressure. Moreover, it plays an important role in autoregulation and recovery from brain injuries [20].

In our group, EtCO_2_ was monitored at both positions. EtCO_2_ decreases of more than 2 mmHg occurred in 35.06% of patients in the sitting position and in 15.66% of patients in the park-bench position. Independent of VAE, fluctuations in EtCO_2_ levels occurred in both positions during patient mobilization for positioning, both before and at the end of surgery.

In the present study, VAE was not documented in the park-bench group, but given the fluctuating EtCO_2_ drops and episodes of hemodynamic instability, transient VAE episodes were suspected. In one study, Himes et al. reported a 12% incidence of VAE in the horizontal position in 74 adults monitored via precordial Doppler [25].

In our sitting group, four patients had hypoxia (1.73%), whereas in the park-bench group, hypoxia was present in seven patients (8.43%). Decreased arterial SpO_2_ could be used to confirm VAE but may not occur despite the onset of VAE if high concentrations of inspired oxygen are used. A shunt increase occurs early during VAE; therefore, inspired oxygen should be increased early in VAE management [26]. Desaturation episodes in both positions have been associated with the occurrence of severe arterial hypotension before vasopressor correction. In the park-bench position, hypoxia episodes are caused by atelectasis and an altered ventilation/perfusion ratio, requiring an increased fraction of inspired oxygen and alveolar recruitment maneuvers during the procedure.

One reason why the sitting position is preferred over the horizontal position is that it reduces intraoperative bleeding. A retrospective review of 579 posterior fossa craniectomies at the Mayo Clinic [9,25] over 3 years revealed that the transfusion requirements were more than 2 units of blood in 13% of patients who underwent an operation in the horizontal position and only 3% of patients with who underwent an operation in the sitting position. In the present study, transfusions were given to 2.6% of the patients in the sitting group and 8.43% of those in the park-bench group.

Patient positioning has an impact on cerebral hemodynamics and, implicitly, on intracranial pressure, particularly in the sitting and park-bench positions. Regardless of the mechanism by which supratentorial pneumocephalus and ventricular air accumulation occur in the sitting position, whether due to the pressure difference between atmospheric air and the skull structures after dura mater incision, during tumor resection through tumor volume reduction or excessive CSF drainage, the cephalic extremity above the heart is the culprit. In the park-bench position, pneumocephalus is less common—the mechanisms of its occurrence are the same, but the difference is that the cephalic extremity is not located more than 20° above the heart, and the intracranial air volume is reduced. The severity of the complication is affected by the intracranial air volume. The supratentorial pneumocephalus diagnosis is performed via postoperative brain computed tomography (CT), with the presence of the characteristic Mount Fiji sign [27]. Sloan, T. et al. described supratentorial pneumocephalus occurrence in 42.1% of patients who underwent an operation in the sitting position; these patients had an air volume between 6 and 280 cm^3^, with potential somatosensory impairment due to cerebral ischemia when the intracranial air volume exceeded 90 cm^3^ [28]. In the present study, only 12 (5.19%) of the patients who underwent surgery in the sitting position and 3 (3.61%) of the patients who underwent surgery in the park-bench position had supratentorial pneumocephalus. None of the patients required a twist drill procedure for evacuation or insertion of an external ventricular drain. Treatment consisted of oxygen therapy (2 L O_2_/min for 4–6 h/day) and chest and cephalic extremity elevation to 40° until complete resorption. Air absorption occurred gradually, starting on the second postoperative day, with dynamic monitoring via native brain CT.

Both cerebral venous pressure decrease, which involves reducing intraoperative hemorrhage and venous clotting time, and postoperative increases in cerebral venous pressure, which are part of the treatment for VAE, increase the risk of postoperative hematoma in patients who undergo operation in the sitting position. In the park-bench position, postoperative hematoma may occur as a result of increased venous pressure in the cerebral hemisphere near the floor plane due to gravity or uneven venous drainage when the venous sinus tears. Regardless of patient position, an important role is played by individual factors, such as associated pathology, preexisting coagulopathy, fluctuating blood pressure values, and tumor vascularization. In the literature, postoperative hematoma is described as having a low risk of occurrence; however, it has devastating effects [1,18,25,29,30]. The present study revealed the presence of postoperative hematoma in the tumor bed of 10 (4.33%) patients who underwent operation in the sitting position and in 6 (7.23%) patients who underwent operation in the park-bench position. Treatment consisted of hematoma evacuation. General preventive measures include careful blood pressure monitoring, rigorous hemostasis, and correct positioning of the patient before and after surgery [31,32].

The influence of neurosurgical patient positioning on hydrocephalus development has rarely been studied. In the sitting position, CSF aspiration contributes to good visibility of the operative field, but prolonged suction may lead to decreased uptake of CSF by the arachnoid villi due to temporary inactivity, resulting in hydrocephalus [33]. Obstruction of the aqueduct of Sylvius following resection of pineal region tumors or midbrain tectal tumors is another cause of hydrocephalus, either intraoperatively or postoperatively [34]. Another triggering factor for hydrocephalus is postoperative mixed CSF cellularity, which may block absorption by the villi, resulting in communicating hydrocephalus [35]. Very rarely, hydrocephalus is caused by ventriculitis due to CSF flow obstruction [36].

In the park-bench position, increased venous pressure in the cerebral hemisphere near the operating table predisposes patients to venous infarction and associated cerebral edema [21,25] and hemodynamic changes with an impact on altering CSF dynamics, resulting in hydrocephalus. Surgical handling of the anatomical structures of the posterior fossa to obtain easy access may alter the free CSF flow. The general factors specific to both neurosurgical positions are the obstruction of CSF flow resulting from tumor debris [37], postoperative hematoma [4], or postoperative scar sites [38]. Preexisting hydrocephalus may be a cause of postoperative hydrocephalus development. Duraplasty or insertion of an external ventricular or ventriculoperitoneal shunt may influence hydrocephalus development either by overdrainage [25] or insufficient drainage [39]. In our study, seven (3.03%) patients developed hydrocephalus in the sitting position, and four (4.82%) developed hydrocephalus in the park-bench position. The treatment used was ventriculocisternostomy (VCS) in three (27.27%) patients and ventriculoperitoneal drainage (VPD) in eight (72.73%) patients. The recommended preventive measures are avoiding excessive CSF drainage, careful dissection of posterior fossa structures and, last but not least, intraoperative imaging of CSF flow to exclude or eliminate obstructive sites. Postoperative imaging follow-up is essential in the management of early hydrocephalus signs.

Cranial nerve injury during neurosurgical procedures may occur due to several factors, and patient positioning may also influence the risk of injury. In the sitting position, cranial nerve injury most commonly occurs through prolonged traction and compression [40]. The facial nerve and trigeminal nerve are frequently affected in posterior fossa surgery [41]. By decreasing cerebral perfusion pressure, which is induced by the sitting position due to gravity, cranial nerves become more susceptible to ischemia during prolonged neurosurgical interventions [8]. In the park-bench position, the accessory and hypoglossal cranial nerves are most frequently affected [42], either due to cephalic extremity hyperextension during positioning or compression of the surgical instruments, given the limited operating field, as gravity does not influence CSF or hemorrhage drainage. The duration of surgery and preexisting cranial nerve damage influence the degree of intraoperative injury and, thus, the postoperative prognosis [21]. Absent or inadequate intraoperative neurophysiological monitoring may increase the risk of cranial nerve injury as early nerve damage signs go unnoticed [43]. In the present study, only seven (3.03%) patients presented House–Brackmann grade VI facial paralysis: four patients who underwent operation in the sitting position and three patients who underwent operation in the park-bench position. Facial paralysis recovery grades of III and IV occurred in two (28.57%) and five (71.43%) patients, respectively. To minimize cranial nerve injury risk, it is necessary to pay special attention to patient positioning, use a surgical technique that allows the surgeon a level of intraoperative comfort, avoid excessive traction or compression, ensure adequate cerebral perfusion, and use neurophysiological monitoring.

In the sitting position, VAE causes hypotension and decreased cerebral perfusion [44], thus increasing the risk of ischemia in the brainstem and cerebellar hemisphere [8]. Decreased central venous pressure may reduce cerebral perfusion and increase cerebellar susceptibility to ischemia, especially when the self-regulatory mechanism is insufficient [45]. Changes in intracranial pressure, either due to hypotension or excessive CSF drainage, compromise blood flow to posterior fossa structures, leading to potential posterior fossa ischemia. In the park-bench position, lateral rotation of the cephalic extremity may cause compression of the posterior inferior cerebellar artery, with the development of ischemia in the cerebellar hemisphere [46]. Because of gravity, the park-bench position can cause a difference in cerebral blood flow and venous return between the two cerebral hemispheres [47], an alteration that predisposes individuals to cerebral perfusion imbalance by compromising cerebellar circulation, especially in the case of increased pressure in the posterior fossa, accelerating the onset of ischemic lesions at this level. Both hypercapnia, caused by respiratory changes following park-bench positioning [48], and hypotension [25], as part of blood pressure fluctuations, can reduce cranial pressure, both of which increase the risk of posterior fossa ischemia. However, general factors specific to both positions should not be overlooked either. These include long surgery durations [47], surgical manipulation, inadequate blood pressure management, and, last but not least, preexisting vascular conditions. In our study, two (0.87%) patients who underwent surgery in the sitting position and one (1.2%) patient who underwent surgery in the park-bench position experienced posterior fossa ischemia. All three patients required decompressive craniectomy, but they unfortunately died. In total, eight deaths occurred: three (1.3%) patients who underwent an operation in the sitting position and five (6.03%) patients who underwent an operation in the park-bench position. The other patient who underwent an operation in the sitting position and the other four patients who underwent an operation in the park-bench position died due to postoperative hematoma.

Early extubation may have negative repercussions on a patient’s neurological and respiratory recovery. Therefore, depending on the events that occurred intraoperatively, the time of extubation is decided between the anesthesiologist and neurosurgeon. Delayed postoperative extubation, prolonged duration of anesthesia and surgery, and the need for prolonged intensive care unit stay lead to an unfavorable prognosis, with the occurrence of respiratory complications that may have a negative impact on neurological status and delay recovery [49,50]. In our study, immediate postoperative extubation was performed in 215 (93.07%) patients who underwent an operation in the sitting position and 72 (86.75%) patients who underwent an operation in the park-bench position. Postoperative ventilation was required for 16 (6.93%) patients who underwent an operation in the sitting position and 11 (13.25%) patients who underwent an operation in the park-bench position. Prolonged postoperative ventilation hinders neurologic evaluation, diagnosis, and therapeutic decision-making, and imaging remains the only available diagnostic method. In both positions, the time spent under anesthesia was approximately the same.

Correct patient positioning, careful intraoperative monitoring, maintenance of hemodynamic stabilization, and careful surgical resection are recommended to prevent the development of posterior fossa ischemia. By understanding the specific risks associated with both the neurosurgical position and the implementation of strategic preventive measures, the possibility of posterior fossa complications can be significantly reduced.

## 6. Limitations of the Study

### 6.1. Limitations

This study, while comprehensive, has several limitations that should be considered. First, its retrospective design inherently limits the ability to establish causal relationships between patient positioning and the observed outcomes. The study relied on historical data, which may include inconsistencies or missing information, potentially affecting the accuracy of the findings. Additionally, the sample size, particularly for the park-bench position group, was relatively small, which may limit the power of the statistical analyses and the generalizability of the results. The single-center nature of the study further restricts the applicability of the findings to other settings with different patient populations and surgical practices. Finally, unmeasured confounders, such as the variability in surgeon experience and patient comorbidities, may have influenced the outcomes but were not fully accounted for in the analysis.

### 6.2. Strengths

Despite these limitations, the study has several strengths that enhance the credibility of its findings. The inclusion of a substantial number of patients over a six-year period provides a robust dataset, allowing for a detailed analysis of the effects of surgical positioning on intraoperative and postoperative outcomes. This study is one of the few to directly compare the sitting and park-bench positions in a neurosurgical setting, providing valuable insights that can inform clinical practice.

### 6.3. Clinical Implications

The findings of this study have important clinical implications for neurosurgical practice. The significantly higher incidence of hypotension and anesthetic complications in patients operated on in the sitting position suggests the need for heightened vigilance and proactive management strategies during such procedures. The identification of specific risk factors, such as CO_2_ decrease and documented air embolism, further underscores the importance of comprehensive intraoperative monitoring and timely intervention to mitigate these risks. On the other hand, the increased incidence of hypoxemia and mortality in the park-bench position highlights the need for careful patient selection and optimization of perioperative care in this group. These insights can guide anesthesiologists and surgeons in tailoring their approach to each patient’s unique risks, ultimately improving surgical outcomes and patient safety.

## 7. Conclusions

The sitting position involves a balance between its unique surgical advantages and the associated risks. Owing to its benefits, the success in managing perioperative complications increases, and the risks associated with positioning can be controlled or minimized without repercussions on the prognosis. Even though the sitting position is a controversial neurosurgical position, this study confirms that it is a viable position and is comparable to other positions. The neurosurgeon may opt for park-bench positioning, with reservations regarding the surgical benefits, as it may prolong the postoperative recovery time. Although compared with the sitting position, the park-bench position may offer greater hemodynamic stability and reduce the risk of anesthetic complications, its use is associated with a potential need for prolonged postoperative ventilation and a higher rate of death. The choice of surgical position should take these risks into consideration and be tailored to each individual patient.

## Figures and Tables

**Table 1:** Patient data.

Variables	Sitting Position (*n* = 231)	Park-Bench Position (*n* = 83)	*p* Value
Age (years), median (IQR)	55 (43–63)	62 (45–74)	<0.001
Sex (F), no. (%)	138 (59.74)	48 (57.83)	0.761
Surgical approach, detailed, no. (%)	Retrosigmoid: 152 (65.8) Unilateral suboccipital: 48 (20.78) Midline: 18 (7.79) Infratentorial supracerebellar: 13 (5.63)	Unilateral suboccipital: 58 (69.8) Retrosigmoid: 22 (26.51) Midline: 2 (2.41) Infratentorial supracerebellar: 1 (1.2)	<0.001
Anesthesia complications, no. (%)	212 (91.77)	59 (71.08)	<0.001
Hypertension (>140/90 mmHg), no. (%)	120 (51.95)	33 (39.76)	0.057
Hypotension (<90/60 mmHg), no. (%)	143 (61.9)	14 (16.87)	<0.001
Tachycardia (>100 bpm), no. (%)	32 (13.85)	18 (21.69)	0.094
Bradycardia (<60 bpm), no. (%)	88 (38.1)	13 (15.66)	<0.001
Atropine, no. (%)	58 (25.11)	7 (8.43)	0.001
Colloid, no. (%)	59 (25.54)	35 (42.17)	0.005
Vasoactive, no. (%)	85 (36.8)	15 (18.07)	0.002
CO_2_ decreases by >2 mmHg, no. (%)	81 (35.06)	13 (15.66)	<0.001
Hypoxia, no. (%)	4 (1.73)	7 (8.43)	0.009
Documented VAE, no. (%)	24 (10.39)	0 (0)	0.002
Aspirated air (mL), median (IQR)	0 (0–0)/ 20 (15–30) {10–60} *	0 (0–0)	0.003
Intraoperative transfusions, no. (%)	6 (2.6)	7 (8.43)	0.047
Intraoperative diuresis (mL), median (IQR)	1628 (1382.5–1901.5)	1566 (1358.5–1859.5)	0.408
Immediate postoperative extubation, no. (%)	215 (93.07)	72 (86.75)	0.078
Anesthesia time (min), median (IQR)	433 (370–473.5)	415 (368–446)	0.057
Total surgery time (min), median (IQR)	348 420 (300–1020) (289–385.5)	331 (287.5–360)	0.058
Postoperative ventilation, no. (%)	16 (6.93)	11 (13.25)	0.078
Postoperative ventilation (min), median (IQR)	0 (0–0)/ 990 (885–1140) {540–2880}	0 (0–0)/ 420 (300–1020) {180–5760}	0.101
Postoperative hematoma, no. (%)	10 (4.33)	6 (7.23)	0.6
Pneumocephalus, no. (%)	12 (5.19)	3 (3.61)	0.767
Hydrocephalus, no. (%)	7 (3.03)	4(4.82)	0.613
Cranial nerve paralysis (total facial paralysis)	7 (3.03)	0 (0.0)	0.196
Ischemic CVA, no. (%)	2 (0.87)	1 (1.2)	1
Death, no. (%)	3 (1.3)	5 (6.03)	0.033

*, aspirated air only for patients who have been aspirated (ignoring 0 values); {} denotes the range.

**Table 2:** Multivariate logistic regression model to predict hypotension based on sitting position, adjusted for CO_2_ decrease, desaturation, and documented VAE.

Characteristics	OR Adjusted	(95% CI)	*p*
Sitting position (sitting vs. park-bench)	7.25	(3.77–14.98)	<0.001
CO_2_ decreases by >2 mmHg (sitting vs. park-bench)	3.37	(1.77–6.66)	<0.001
Hypoxia (sitting vs. park-bench)	1.23	(0.28–5.67)	0.786
Documented VAE (sitting vs. park-bench)	1.08	(0.36–3.68)	0.89

OR, odds ratio; CI, confidence interval.

## Data Availability

The original contributions presented in this study are included in the article. Further inquiries can be directed to the corresponding author.

## References

[1] Lubnin A.Y. (2022). Neirokhirurgicheskie vmeshatel’stva v polozhenii sidya: Real’no otsenivaya riski [Sitting position in neurosurgery: Realizing the risks]. Vopr. Neirokhirurgii Im. N. N. Burdenko.

[2] Welch M.B., Wahr J.A., Crowley M. (2018). Patient Positioning for Surgery and Anesthesia in Adults. UpToDat. https://medilib.ir/uptodate/show/94593.

[3] Domaingue C.M. (2005). Anaesthesia for neurosurgery in the sitting position: A practical approach. Anaesth. Intensive Care.

[4] Emamimeybodi M., Hajikarimloo B., Abbasi F., Tavanaei R., Toudeshki K.K., Koohi N., Pourhemmati S., Amani H., Pishgahi M., Oraee-Yazdani S. (2024). Position-dependent hemodynamic changes in neurosurgery patients: A narrative review. Interdiscip. Neurosurg..

[5] Elton R.J., Howell R.S. (1994). The sitting position in neurosurgical anaesthesia: A survey of British practice in 1991. Br. J. Anaesth..

[6] Kida H., Nishikawa N., Matsunami K., Kawahito M., Ota M., Miyao S. (2000). Sitting position in the neurosurgery: The results of a questionnaire sent to neurosurgeons of medical colleges. Masui.

[7] Macalinao A.B., Gavia L.A. (2023). A Comparison of Intraoperative Occurrence of Venous Air Embolism During Posterior Fossa Surgeries in Adult Patients in Sit Up vs Horizontal Position: A Systematic Review and Meta-Analysis Study.

[8] Kore S. (2016). A prospective study observing outcome following posterior fossa craniotomy in patients with sitting position. Int. J. Biomed. Adv. Res..

[9] Saladino A., Lamperti M., Mangraviti A., Legnani F.G., Prada F.U., Casali C., Caputi L., Borrelli P., DiMeco F. (2017). The semisitting position: Analysis of the risks surgical outcomes in a contemporary series of 425 adult patients undergoing cranial surgery. J. Neurosurg..

[10] Lindroos A.-C., Niiya T., Randell T., Romani R., Hernesniemi J., Niemi T. (2010). Sitting Position for Removal of Pineal Region Lesions: The Helsinki Experience. World Neurosurg..

[11] Dallier F., Di Roio C. (2015). Sitting position for pineal surgery: Some anaesthetic considerations. Neurochirurgie.

[12] Biaggioni I. (2018). Orthostatic Hypotension in the Hypertensive Patient. Am. J. Hypertens..

[13] Joseph A., Wanono R., Flamant M., Vidal-Petiot E. (2017). Orthostatic hypotension: A review. Nephrol. Ther..

[14] Mar P.L., Raj S.R. (2018). Orthostatic hypotension for the cardiologist. Curr. Opin. Cardiol..

[15] Waxenbaum J.A., Reddy V., Varacallo M. (2023). Anatomy, autonomic nervous system. StatPearls.

[16] Ricci F., De Caterina R., Fedorowski A. (2015). Orthostatic Hypotension: Epidemiology, Prognosis, and Treatment. J. Am. Coll. Cardiol..

[17] Allman K., Wilson I. (2017). Oxford. Practical Guide to Anesthesia.

[18] Iavarone I.G., Rocco P.R.M., Silva P.L., Taran S., Wahlster S., Schultz M.J., Robba C. (2024). Perioperative Ventilation in Neurosurgical Patients: Considerations and Challenges. Curr. Anesthesiol. Rep..

[19] Günther F., Frank P., Nakamura M., Hermann E.J., Palmaers T. (2017). Venous air embolism in the sitting position in cranial neurosurgery: Incidence and severity according to the used monitoring. Acta Neurochir..

[20] Prabhakar H., Mahajan C., Kapoor I. (2017). Manual of Neuroanesthesia. The Essentials.

[21] Dilmen O.K., Akcil E.F., Tureci E., Tunali Y., Bahar M., Tanriverdi T., Aydin S., Yentur E. (2011). Neurosurgery in the sitting position: Retrospective analysis of 692 adult and pediatric cases. Turk. Neurosurg..

[22] Konrad F.M., Konrad F.M., Mayer A.S., Mayer A.S., Serna-Higuita L.M., Serna-Higuita L.M., Hurth H., Hurth H., Tatagiba M., Tatagiba M. (2022). Occurrence and severity of venous air embolism during neurosurgical procedures: Semisitting versus supine position. World Neurosurg..

[23] Vacas S., Van de Wiele B. (2017). Designing a pain management protocol for craniotomy: A narrative review and consideration of promis ing practices. Surg. Neurol. Int..

[24] Connor C.W. (2020). A forensic disassembly of the BIS monitor. Anesth. Analg..

[25] Himes B.T., Mallory G.W., Abcejo A.S., Pasternak J., Atkinson J.L.D., Meyer F.B., Marsh W.R., Link M.J., Clarke M.J., Perkins W. (2016). Contemporary analysis of the intraoperative perioperative complications of neurosurgical procedures performed in the sitting position. J. Neurosurg..

[26] Brull S.J., Prielipp R.C. (2017). Vascular air embolism: A silent hazard to patient safety. J. Crit. Care..

[27] Hanalioglu D., Elbir C., Sahin O.S., Ercandirli A.K., Sahin B., Turkoglu M.E., Kertmen H.H., Hanalioglu S. (2023). Clinical Significance of Pneumocephalus in Pediatric Mild Traumatic Brain Injury. Pediatr. Emerg. Care.

[28] Choque-Velasquez J., Colasanti R., Resendiz-Nieves J.C., Gonzáles-Echevarría K.E., Raj R., Jahromi B.R., Goehre F., Lindroos A.-C., Hernesniemi J. (2018). Praying sitting position for pineal region surgery: An efcient variant of a classic position in neurosurgery. World Neurosurg..

[29] Mathur A. (2022). Abstract No.: ABS0480: A retrospective study observing outcome following posterior fossa craniotomy in patients with sitting position. Indian J. Anaesth..

[30] Moerman A., De Hert S. (2015). Cerebral oximetry: The standard monitor of the future?. Curr. Opin. Anesthesiol..

[31] Suarjaya I.P.P., Paramartha B., Sutawan I.B.K.J., Panji I.P.A.S. (2022). Manajemen Anestesi Reseksi Tumor Cerebello-pontine Angle Vestibular Schwannoma dengan Posisi Lateral. J. Neuroanestesi Indones..

[32] Mavarez-Martinez A., Israelyan L.A., Soghomonyan S., Fiorda-Diaz J., Sandhu G.S., Shimansky V.N., Ammirati M., Palettas M., Lubnin A.Y., Bergese S.D. (2020). The Effects of Patient Positioning on the Outcome During Posterior Cranial Fossa and Pineal Region Surgery. Front. Surg..

[33] Barami K. (2016). Cerebral venous overdrainage: An under-recognized complication of cerebrospinal fluid diversion. Neurosurg. Focus.

[34] Avagliano L., Massa V., George T.M., Qureshy S., Bulfamante G.P., Finnell R.H. (2019). Overview on neural tube defects: From development to physical characteristics. Birth Defects Res..

[35] Magnaes B. (1978). Movement of cerebrospinal fluid within craniospinal space when sitting up and lying down. Surg. Neurol..

[36] Muzumdar D.P. (2020). Postoperative pneumoventricle following posterior fossa tumor surgery in sitting position: Plugging the aqueduct. J. Pediatr. Neurosci..

[37] Taki K., Ninomiya K., Yamamoto A., Suematsu T., Sasaki M., Kishima H. (2024). Communicating hydrocephalus after resection of a meningioma ventral to the foramen magnum. Surg. Neurol. Int..

[38] Motiei-Langroudi R., Griessenauer C.J., Alturki A.Y., Chapman P.H., Ogilvy C.S., Thomas A.J. (2017). Modified Park Bench Position for Superior Vermian Arteriovenous Malformations and Dural Fistulas. World Neurosurg..

[39] Koueik J., Iskandar B.J., Yang Z., Kraemer M.R., Armstrong S.A., Wakim V., Broman A.T., Medow J.E., Luzzio C., Hsu D.A. (2021). Ventriculoperitoneal Shunt Drainage Increases with Gravity and Cerebrospinal Fluid Pressure Pulsations: Benchtop Model. Neurosurgery.

[40] Charbel F.T., Kehrli P., Pain L. (1998). The sitting position in neurosurgery: The viewpoint of the surgeon. Ann. Fr. D’Anesth. Reanim..

[41] Schaffranietz L., Günther L. (1997). Die sitzende Position bei Operationen in der Neurochirurgie. Ergebnisse einer Umfrage [The sitting position in neurosurgical operations. Results of a survey]. Anaesthesist.

[42] Gunerhan G., Cagil E., Daglar Z., Aygun K., Yigit M., Algul H., Belen A.D. (2024). Surgical management of foramen magnum meningiomas: Clinical insights and outcomes. Ann. Med. Res..

[43] Bilotta F., Sergi P.G., Spennati V. (2020). Patient Positioning during Neurosurgery: A Relevant Skill for Neuroanesthesiologist in a Multidisciplinary Team Work. J. Neuroanaesth. Crit. Care.

[44] Khan Z.H., Samadi S., Zanjani A.P., Hatemi B.M. (2019). Air Embolism in Sitting Position during Neurosurgical Operations and It’s Prevention: A Narrative Review. Arch. Anesth. Crit. Care..

[45] Pinto V.L., Tadi P., Adeyinka A. (2024). Increased Intracranial Pressure. StatPearls.

[46] Haines D.E., Haines D.E., Mihailoff G.A. (2018). Chapter 8—A Survey of the Cerebrovascular System. Fundamental Neuroscience for Basic and Clinical Applications.

[47] Babakhani B., Schott M., Hosseinitabatabaei N., Jantzen J.P. (2015). Unanticipated Disturbance in Somatosensory Evoked Potentials in a Patient in Park-Bench Position. Turk. J. Anaesthesiol. Reanim..

[48] Yahanda A.T., Chicoine M.R. (2020). Paralysis caused by spinal cord injury after posterior fossa surgery: A systematic review. World Neurosurg..

[49] Cinotti R., Bruder N., Srairi M., Paugam-Burtz C., Beloeil H., Pottecher J., Geeraerts T., Atthar V., Guéguen A., Triglia T. (2018). Prediction Score for Postoperative Neurologic Complications after Brain Tumor Craniotomy: A Multicenter Observational Study. Anesthesiology.

[50] Song G., Liu D., Wu X., Wang X., Zhou Y., Li M., Lin Q., Guo H., Tang J., Xiao X. (2021). Outcomes after semisitting and lateral positioning in large vestibular schwannoma surgery: A single-center comparison. Clin. Neurol. Neurosurg..

